# Extraction, Structural Characterization, and Physicochemical and Biological Properties of Water-Soluble Polysaccharides from Adlay Bran

**DOI:** 10.3390/molecules29194707

**Published:** 2024-10-04

**Authors:** Peng Hu, Guangjing Chen

**Affiliations:** 1 School of Pharmacy, Hunan Traditional Chinese Medical College, Zhuzhou 412012, China; hupeng2007@163.com; 2 College of Food Science and Engineering, Guiyang University, Guiyang 550005, China

**Keywords:** polysaccharides, adlay bran, structure, chemical structure, physicochemical characterization, antioxidant activity, hypoglycemic activity

## Abstract

Adlay bran, often discarded or used as animal feed, holds untapped potential. This study explores the beneficial properties of water-soluble polysaccharides (ABPs), extracted using a hot water method, with the aim of transforming what is commonly regarded as waste into a valuable resource. The response surface methodology (RSM) was employed to fine-tune the extraction parameters, establishing conditions at 80.0 °C, 2.5 h, and a water-to-material ratio of 31.6 mL/g. Structural studies showed that ABPs consist of different monosaccharides, including rhamnose, arabinose, glucosamine, glucose, galactose, xylose, mannose, and glucuronic acid, with respective molar ratios of 2.12%, 2.40%, 0.52%, 77.12%, 7.94%, 3.51%, 2.55%, and 3.82%. The primary component of these polysaccharides has a molecular weight averaging 12.88 kDa. The polysaccharides feature eight distinct linkage types: →3,4)-Rha*p*-(1→ at 5.52%, →4)-Glc*p*-(1→ at 25.64%, Glc*p*-(1→ at 9.70%, →3,4)-Glc*p*-(1→ at 19.11%, →4)-Xyl*p*-(1→ at 7.05%, →3)-Glc*p*-(1→ at 13.23%, →3,4)-Gal*p*-(1→ at 9.26%), and →4,6)-Gcl*p*-(1→ at 12.49%. The semi-crystalline properties of ABPs and their shear-thinning characteristics were validated by X-ray diffraction and rheology tests. In vitro assays highlighted the strong antioxidant activities of ABPs, as evidenced by DPPH and ABTS hydroxyl radical scavenging tests, along with significant metal chelating and reducing powers. Additionally, ABPs showed significant inhibition of α-glucosidase and α-amylase, making them attractive as versatile additives or as agents with antioxidant and blood-sugar-lowering properties in both the food and pharmaceutical sectors. These findings support the utilization of adlay bran for higher-value applications, harnessing its bioactive components for health-related benefits.

## 1. Introduction

Polysaccharides, essential biomacromolecules, are present in the cells of animals, plants, and microorganisms. Primarily extracted from plants, microorganisms, and marine organisms, these substances exhibit diverse bioactivities, including antioxidant, anticoagulant, immunomodulatory, and antidiabetic effects [1,2,3]. Due to their strong hydrophilic properties, excellent rheological attributes, beneficial bioactivities, and non-toxic nature, plant-derived polysaccharides are increasingly preferred in functional food, biomedical, and pharmaceutical sectors [4,5,6]. However, the exploration of polysaccharides is complicated by their structural diversity, which is influenced by the variety of raw materials used in extraction. This diversity, reflected in variations in molecular weight, monosaccharide composition, and glycosidic bond configurations, directly affects their biological effectiveness and functional properties [7,8]. Therefore, comprehensive characterization of polysaccharides from novel sources, focusing on their structural details, functional benefits, and biological effects, is crucial for evaluating their potential applications in healthcare, food production, and pharmaceutical industries.

Adlay (*Coix lacryma-jobi* L. var. *ma-yuen* Stapf.) seeds, commonly known as *Coix* seeds or Job’s tears, are important members of the Gramineae family. These seeds thrive in the tropical and subtropical climates of East and Southeast Asia, with Guizhou Province, China, being a notable cultivation area [9]. Valued in traditional Chinese medicine for their medicinal and nutritional properties, adlay seeds are rich in various bioactive compounds, including polysaccharides, phenols, flavonoids, lipids, proteins, fibers, vitamins, oils, alkaloids, steroids, and lactams [10,11]. Known as “Green Food”, adlay is celebrated for its health benefits, including anti-inflammatory, anti-cancer, anti-allergic, and antioxidant properties, as well as its ability to modulate gut microbiota [12,13,14]. Processing adlay seeds yields a significant amount of adlay bran, comprising about 25% of the seed’s dry weight. This by-product, rich in phenolics, polysaccharides, neutral oils, and dietary fibers, exhibits various biological activities, including antioxidant, anti-inflammatory, anti-cancer, antiviral, and antimicrobial properties [14,15,16]. Despite its valuable properties, adlay bran is often underutilized, mainly used as animal feed or discarded, which not only underestimates its potential but also creates environmental concerns. Given its rich nutritional and bioactive profile, adlay bran has significant potential as an economic resource in the food industry. Developing innovative uses for adlay seed bran is essential to fully harness its benefits. Extensive research to date has focused on the dietary fiber and phenolics in adlay bran [17,18]. However, only a few studies have explored the potential of adlay bran polysaccharides in mitigating TNF-α-induced epithelial barrier dysfunction [19]. Although adlay bran is recognized as a significant source of water-soluble polysaccharides, knowledge about their structural characteristics, functional properties, and antioxidant and hypoglycemic effects remains limited. This gap highlights the need for more comprehensive studies to unlock the full potential of adlay bran in various applications.

Therefore, the aim of this research was to isolate adlay bran polysaccharides (ABPs) and investigate their physicochemical characteristics, rheological properties, and in vitro antioxidant and hypoglycemic activities. The research began by creating an enhanced extraction process for ABPs, utilizing a hot water extraction technique, which was improved through individual variable tests and the response surface methodology (RSM). After extraction, the structural and physicochemical properties of the ABPs were thoroughly examined using a range of methods such as colorimetry, high-performance gel permeation chromatography (HPGPC), high-performance anion-exchange chromatography (HPAEC), gas chromatography–mass spectrometry (GC-MS), Fourier transform infrared spectroscopy (FT-IR), X-ray diffraction (XRD), scanning electron microscopy (SEM), atomic force microscopy (AFM), thermogravimetric analysis (TGA), and rheometry. The antioxidant and blood-sugar-lowering properties of the ABPs were assessed in vitro using multiple tests, such as DPPH, ABTS, and hydroxyl radical scavenging assays, along with iron (Fe^2+^) binding and inhibition of α-amylase and α-glucosidase activities. The findings of this study are expected to offer valuable insights into the utilization of natural polysaccharides from adlay bran as functional food ingredients or pharmaceutical supplements. By exploring the potential applications of ABPs, this research could pave the way for innovative uses of adlay bran, transforming it from a waste product into a key component of health-promoting products.

## 2. Results and Discussion

### 2.1. Effect of Single Factors on the Yield of ABPs

**Figure 1:**
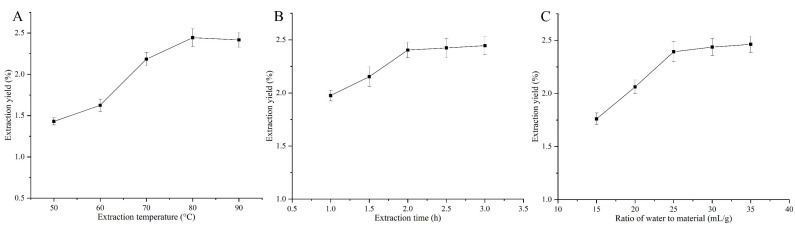
Effects of different extraction temperatures (**A**), extraction times (**B**), and ratios of water to material (**C**) on extraction yield of ABPs.

The effect of the extraction temperature on the ABP yield was investigated while keeping a constant extraction time of 3 h and a water-to-material ratio of 30 mL/g. As shown in Figure 1A, the ABP yield significantly increased as the temperature rose from 50 °C to 80 °C, reaching a maximum of 2.44% at 80 °C. Beyond this point, a slight decrease in yield was observed as the temperature continued to rise, although these changes were not statistically significant (*p* > 0.05). Therefore, 80 °C was selected as the optimal extraction temperature for further optimization using the BBD.

The impact of the extraction duration on polysaccharide production was assessed at a steady temperature of 80 °C with a water-to-material ratio of 30 mL/g. Figure 1B illustrates that the ABP yield surged quickly when the extraction duration was lengthened from 1 h to 2.5 h. Beyond 2.5 h, the yield stabilized, showing no notable variations (*p* > 0.05) with further extended extraction periods. Consequently, a 2.5 h extraction period was chosen as the ideal time for the BBD optimization process.

The impact of varying the water-to-material ratio on the ABP yield was examined, keeping the extraction temperature constant at 80 °C and the extraction duration at 3 h. Figure 1C illustrates that the ABP yield rose markedly when the water-to-material ratio increased from 15 mL/g to 30 mL/g, achieving its peak yield at 30 mL/g. Further increases in the ratio did not result in significant changes in yield (*p* > 0.05). Consequently, a 30 mL/g water-to-material ratio was determined as the focal point for the BBD optimization.

### 2.2. Optimization of Extraction Conditions for ABPs Using BBD

Leveraging the results from the single-variable tests, the extraction parameters for ABPs were refined using a Box–Behnken design. This approach effectively explored the interactions among the three key variables affecting the ABP yield and identified their optimal levels. This study’s experimental setup included 17 trials, incorporating five central points to calculate the pure error sum of squares. To reduce the influence of unpredictable variables, the tests were performed in a random sequence. Table 1 outlines the coded factor levels and actual values for the three variables, providing a structured basis for the optimization process. A second-order polynomial model was developed to predict the extraction yield of ABPs (*Y*), as shown in Equation (1).

(1) $$Y=\;2.54\;+\;0.035X1\;-0.008X2\;+\;0.078X3\;-\;0.060X1X2\;-\;0.085X1X3\;+\;0.187X2X3\;-\;0.499X_{1}^{2}-\;0.317X_{2}^{2}-\;0.147\;X_{3}^{2}$$

**Figure 2:**
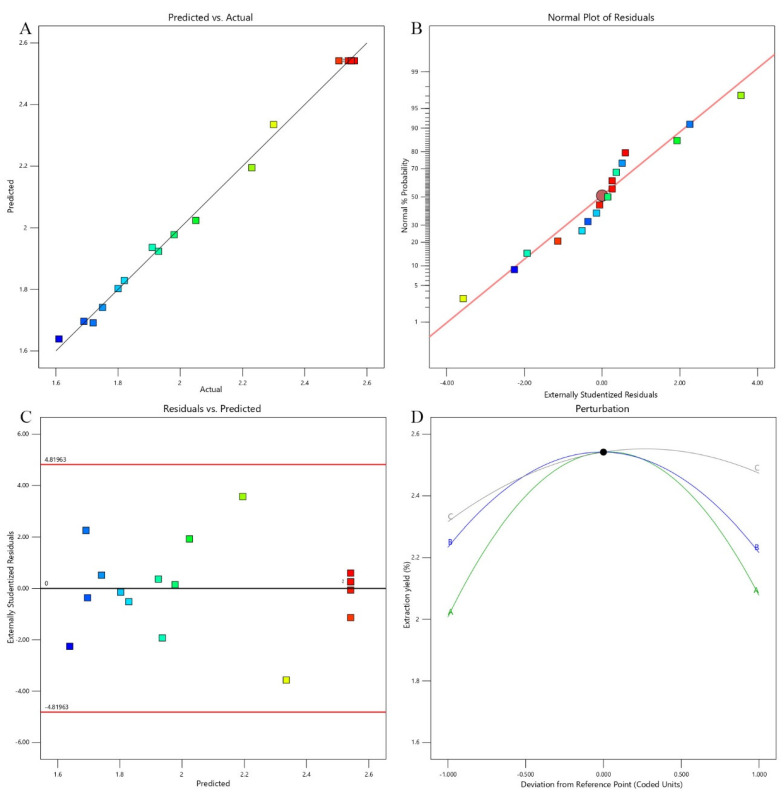
Validation and diagnostic plots of extraction yield under optimal conditions for ABPs: (**A**) comparison of the actual extraction yield with the predicted yield under optimal conditions; (**B**) normal probability plot of internally studentized residuals; (**C**) plot of internally studentized residuals versus predicted response; (**D**) perturbation diagrams illustrating the influences of various factors on the yield. In subfigure (**D**), (A, B, and C) represent the extraction temperature (X_1_), extraction time (X_2_), and ratio of water to material (X_3_), respectively.

Table 2 displays the ANOVA results for the polysaccharide yield response variable. The model’s *F*-value of 210.61 (*p* < 0.0001) indicates that it is highly significant and a strong predictor of the ABP yield. The lack-of-fit *F*-value of 5.16, with a *p*-value of 0.0735, suggests that the model fit is adequate and not significantly influenced by random error [20], confirming its reliability. The model exhibited a substantial *R*^2^ value of 0.9963, signifying a robust relationship between the observed and forecasted yields. This was further supported by an adjusted R^2^ (*R*^2^_Adj_) of 0.9916 and a predicted *R*^2^ (*R*^2^_Pred_) of 0.9520, validating the model’s accuracy and applicability. Figure 2A shows a strong alignment between observed and predicted values, confirming the model’s validity. A coefficient of variation (C.V.) of just 1.54% demonstrates the model’s high consistency. Additionally, the residual normal probability plot (Figure 2B) and the biochemical residual plot (Figure 2C) indicate that the residuals are normally distributed and randomly dispersed around zero, confirming the model’s precision and robustness [21]. The perturbation plot (Figure 2D) reveals that the water-to-material ratio has the greatest impact on ABP yield. This finding aligns with the ANOVA results, further supporting the model’s conclusions. The results demonstrate the practical utility of the optimized conditions in enhancing the efficiency of polysaccharide extraction from adlay bran. The importance of each model’s coefficient was evaluated through *p*-values derived from an *F*-test, where *p*-values under 0.05 signified statistical relevance. Lower *p*-values indicate a more substantial impact of the corresponding variable. Table 2 indicates that the variables *X_1_*, *X_3_*, *X_1_X_2_*, *X_1_X_3_*, *X_2_X_3_*, *X_1_*^2^, *X_2_*^2^, and *X_3_*^2^ had significant coefficients (*p* < 0.05). According to the *F*-values of the linear coefficients, the impact of the three variables on ABP yield is ordered as follows: water-to-material ratio, extraction temperature, and extraction duration.

**Figure 3:**
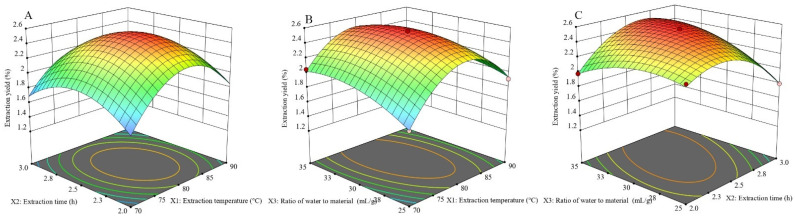
Three-dimensional response surface plots for the interaction effect: (**A**) extraction temperature and extraction time; (**B**) extraction temperature and ratio of water to material; (**C**) extraction time and ratio of water to material.

Three-dimensional response surface plots are used to clarify the interactions between variables and show how response levels change with varying conditions. Figure 3A demonstrates how varying the temperature and duration of extraction impacts the yield. Initially, increasing both temperature and time enhances the extraction yield, but a decline occurs when temperatures rise above 80.34 °C and times exceed 2.53 h. Figure 3B examines the impact of the extraction temperature in combination with the water-to-material ratio, while maintaining a constant extraction time, and identifies an optimal extraction rate at 80 °C with a 30.53 mL/g ratio. On the other hand, Figure 3C highlights the relationship between extraction duration and the water-to-material ratio, pinpointing the maximum extraction yield at 2.55 h and a ratio of 30.78 mL/g. This study indicates that the optimal parameters for ABP extraction are a temperature of 80.03 °C, a duration of 2.54 h, and a water-to-material ratio of 31.58 mL/g, which is expected to yield up to 2.56% ABPs. In practice, these settings were modified to an extraction temperature of 80.0 °C, an extraction duration of 2.5 h, and a water-to-material ratio of 31.6 mL/g. The real experimental outcomes, showing an extraction rate of 2.61 ± 0.07%, did not exhibit any significant statistical difference (*p* > 0.05). The alignment between forecasted and observed outcomes validates the effectiveness and precision of the response model in enhancing the ABP extraction procedure.

### 2.3. Structural Characteristics of ABPs

#### 2.3.1. Chemical and Monosaccharide Composition and Analysis

**Figure 4:**
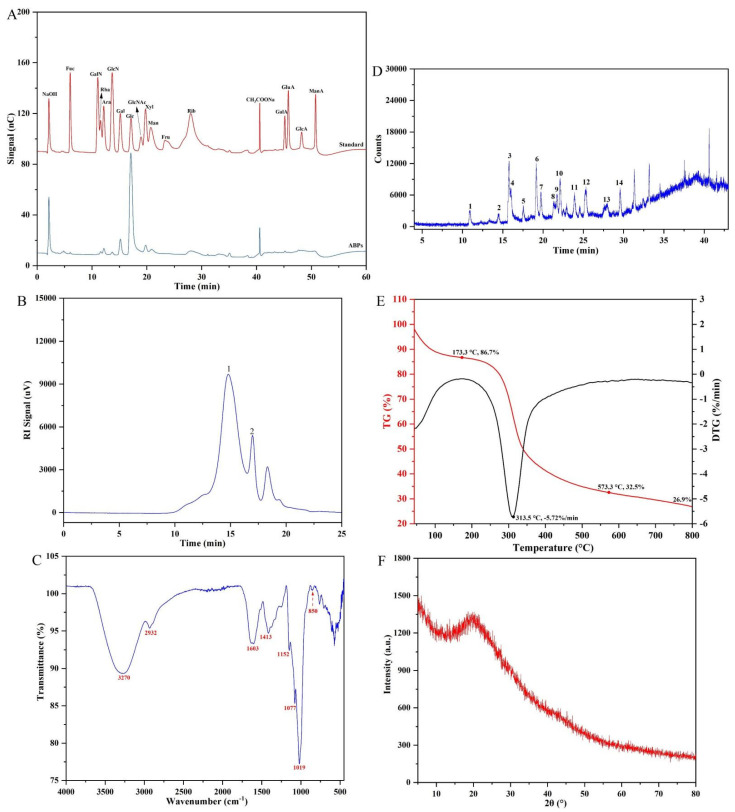
Structural characteristics of ABPs: (**A**) HPAEC chromatograms of ABPs and monosaccharide standards; (**B**) HPGPC chromatogram; (**C**) FT-IR spectra; (**D**) GC-MS chromatogram of PMAAs; (**E**) TG and DTG thermogram curves; (**F**) XRD spectra.

Chemical analysis showed that ABPs mainly consist of 84.12% neutral sugars, 3.03% uronic acids, and 2.32% proteins, as shown in Table 3. This composition closely resembles that of previously reported adlay bran polysaccharides, which contained 88.79% neutral sugars [19]. Because of the presence of uronic acids, ABPs are classified as acidic heteropolysaccharides. As shown in Figure 4A, glucose is the most abundant monosaccharide in ABPs, accounting for 77.12 mol%. Other monosaccharides, including rhamnose, arabinose, glucosamine, galactose, xylose, mannose, and glucuronic acid, are also present, with molar percentages of 2.12, 2.40, 0.52, 7.94, 3.51, 2.55, and 3.82, respectively (Table 3), indicating a complex heteropolysaccharide profile. This monosaccharide composition significantly differs from that of adlay seed, which contains mannose, ribose, glucosamine, galactose, glucose, and arabinose, highlighting the unique structural complexity of ABPs [13].

#### 2.3.2. Molecular Weight

The molecular weight distribution of ABPs was analyzed using HPGPC, as shown in Figure 4B, revealing two distinct peaks, which highlight the inhomogeneity of ABPs. A calibration curve was developed using pullulan polysaccharide standards, resulting in the regression equation LogMw = −0.593x + 12.156, with an *R*^2^ of 0.9854, where ‘x’ denotes the retention time. According to this calibration, the primary peak of ABPs corresponds to an average molecular weight of 12.88 kDa, while the secondary peak reflects a molecular weight of approximately 2.31 kDa, as shown in Table 3. Comparative studies indicate that adlay seed polysaccharides have an Mw of 30.5 kDa [13]. The biological efficacy of polysaccharides is often influenced by their molecular weight, functional groups, and structural arrangements [22]. Differences in Mw between adlay seed and adlay bran polysaccharides likely arise from their different sources.

#### 2.3.3. FT-IR Spectrum

The FT-IR spectrum of ABPs, shown in Figure 4C, displays several notable absorption peaks. A prominent and broad peak at 3270 cm^−1^ represents the O–H bond stretching vibrations, occurring either between or within the polysaccharide chains. The peak at 2932 cm^−1^ is typical of C–H stretching vibrations [23]. The absorption at 1603 cm^−1^ corresponds to C=O stretching vibrations associated with carbonyl groups, while the peak at 1413 cm^−1^ is attributed to the bending vibrations of C–H groups [24]. Additionally, the bands at 1152, 1077, and 1019 cm^−1^ are indicative of pyranose monosaccharide rings [25], while the peak at 850 cm^−1^ confirms the presence of *β*-pyranoside bonds within the polysaccharide structure [26].

#### 2.3.4. Methylation and GC–MS Analysis

To further elucidate the structural features of ABPs, linkage patterns and sugar residue compositions were analyzed using methylation followed by GC-MS analysis. The findings, as shown in Table 4, revealed that ABPs exhibit eight distinctive substitution patterns, benchmarked against the CCRC Spectral Database for PMAAs. These patterns are →3,4)-Rha*p*-(1→ at 5.52%, →4)-Glc*p*-(1→ at 25.64%, Glc*p*-(1→ at 9.70%, →3,4)-Glc*p*-(1→ at 19.11%, →4)-Xyl*p*-(1→ at 7.05%, →3)-Glc*p*-(1→ at 13.23%, →3,4)-Gal*p*-(1→ at 9.26%), and →4,6)-Gcl*p*-(1→ at 12.49%. These data corroborate the monosaccharide composition analysis, confirming that ABPs primarily consist of glucans. The negligible presence of mannose and arabinose signals likely indicates their low concentrations in ABPs.

#### 2.3.5. Thermal Stability and Crystal Structure Analysis

As shown in Figure 4E, thermogravimetric (TG) and derivative thermogravimetric (DTG) analyses were conducted to evaluate the thermal stability of ABPs over a temperature range of 30–800 °C. The analyses revealed a three-stage decomposition pattern. Initially, a weight loss of 13.3% occurred between 50 and 235 °C, attributed to the evaporation of free and bound water in the polysaccharides. In the second phase, spanning 230 to 360 °C, decomposition primarily involved the cleavage of glycosidic bonds and degradation of polysaccharide side chains [27], peaking at a decomposition rate of 5.72%/min at 313 °C. The final phase involved the further breakdown of robust structures, including the cleavage of C–O bonds and glycosidic linkages [28], resulting in a total mass loss of 73.1%. These findings confirm the thermal resilience of ABPs, making them suitable for applications requiring exposure to high temperatures. Furthermore, the molecular structure of ABPs was analyzed using XRD, as shown in Figure 4F. The XRD pattern revealed a broad peak at a diffraction angle of 20.31°, suggesting that ABPs are predominantly amorphous. This absence of crystallinity indicates molecular disarray within ABPs, potentially affecting their biomedical contexts.

#### 2.3.6. SEM Analysis

The surface structure of ABPs, shown in Figure S1 (Supplementary Materials), exhibits a dense and flaky morphology, punctuated by some debris, with a relatively smooth surface and stable configuration. This morphology suggests potential molecular repulsion within the polysaccharide polymers. Such interactions could significantly influence the arrangement and stability of the molecular structure. Understanding these structural characteristics is crucial for exploring the interactions and functional properties of these polymers across different applications, providing insights into their behavior and potential manipulations in various environments.

#### 2.3.7. AFM Analysis

AFM is an invaluable tool for investigating the surface morphology of polysaccharides, allowing precise imaging and functional analysis of biological macromolecules [29]. Figure S2 illustrates that the surface topography of ABPs exhibits molecular dispersion with irregular protrusions, varying in height from −3.9 to 8.1 nm. In aqueous solutions, ABPs predominantly form irregularly aggregated particles rather than linear molecular chains. This is evidenced by a maximum height of approximately 8.1 nm, which exceeds the typical height of 0.1 to 1 nm for individual polysaccharide chains [30]. These observations indicate that the sugar chains within these polysaccharides aggregate in water, resulting in the formation of polymer particles through intermolecular cross-linking.

### 2.4. Rheological Properties

**Figure 5:**
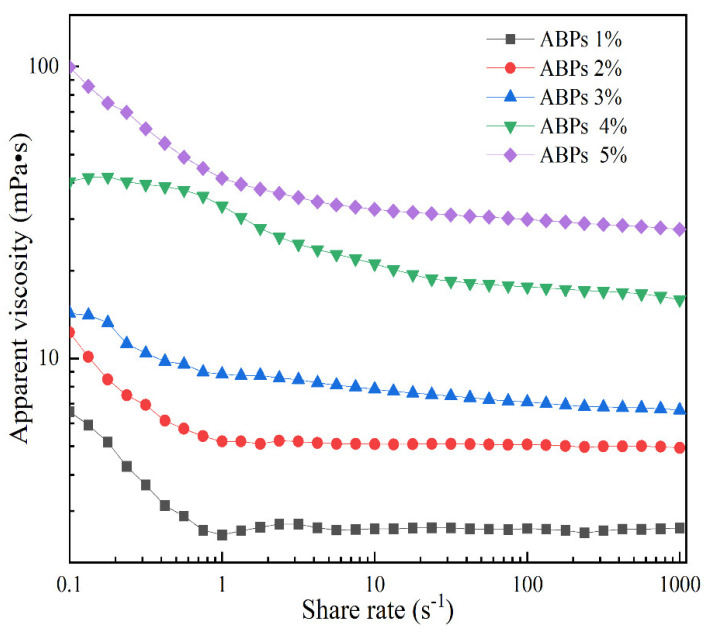
Apparent viscosities of different concentrations of ABPs with the shear rate.

Figure 5 shows the rheological behavior of ABPs, plotting apparent viscosity against shear rate for concentrations ranging from 1% to 5% (*w*/*w*). Across a shear rate range of 0.01 to 1000 s^−1^, all samples displayed shear-thinning behavior, characteristic of non-Newtonian pseudoplastic properties. This pattern, in which viscosity decreases with an increasing shear rate, highlights the colloidal nature of these solutions. Notably, the apparent viscosity of ABPs increases with concentration at a constant shear rate—a characteristic behavior of polymer solutions. As the shear rate increases, the bonding between molecular chains weakens, affecting both molecular weight and the branched structures of the polysaccharides, ultimately reducing apparent viscosity and intermolecular forces, allowing the molecules to adjust to a more compact state [31,32].

The rheological data were analyzed using the Cross (Equation (4)) and Carreau (Equation (5)) models to explore their physical significance and flow characteristics. The statistical parameters, shown in Table 5, demonstrate that the Cross model offers a better fit, as evidenced by the regression coefficients (*R*^2^), effectively capturing the flow behavior of ABP solutions. The ($\eta_{0}$) increased markedly from 0.0074 to 21.2648 Pa·s, and *a* escalated from 4.1190 to 16.6411 s as the ABP concentration increased from 1% to 5%. Conversely, the *d* decreased from 0.7669 to 0.2868 as the concentration increased, showing a strong positive correlation with the concentration and an inverse correlation with the shear rate. As outlined above, the apparent viscosities of ABP solutions are influenced by both the concentration and shear rate. Specifically, viscosity shows a positive correlation with the concentration, increasing as the concentration rises, and an inverse relationship with the shear rate, decreasing as the shear rate increases. This dynamic further illustrates the complex rheological behavior typical of non-Newtonian pseudoplastic materials in ABPs.

### 2.5. Antioxidant Activity of ABPs

**Figure 6:**
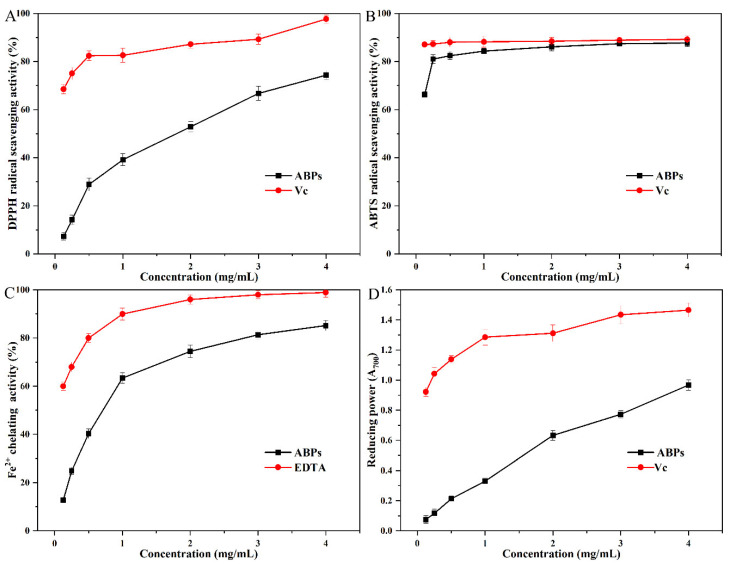
In vitro antioxidant activities of ABPs at different concentrations: (**A**) DPPH radical scavenging activity; (**B**) ABTS radical scavenging activity; (**C**) Fe^2+^ chelating activity; (**D**) reducing power.

Figure 6A illustrates that ABPs exhibited notable scavenging activity against DPPH radicals, with their effectiveness enhancing as concentration increased. The connection between ABPs’ DPPH radical scavenging activity (*y*) and their concentration (*x*) was modeled by the quadratic equation *y* = −4.09*x*^2^ + 33.25*x* + 5.88, showing a significant trend (*p* < 0.05) with an *R*^2^ of 0.977. The scavenging activity increased from 14.20% at 0.25 mg/mL to 74.30% when the concentration reached 4 mg/mL. Despite the fact that the IC_50_ value for ABPs was measured at 1.512 mg/mL, significantly exceeding the IC_50_ of vitamin C (0.030 mg/mL), ABPs nonetheless exhibited strong DPPH radical scavenging properties. This indicates that ABPs are potent free radical inhibitors, highlighting their potential utility in applications that require antioxidant capabilities.

As shown in Figure 6B, ABPs demonstrated significant ABTS radical scavenging activity that intensified with increasing concentrations. When the concentration was 4 mg/mL, ABPs demonstrated a scavenging efficiency of 87.68%, closely approaching the 89.18% efficiency of the positive control, vitamin C, at the identical concentration. The IC_50_ of ABPs was measured at 0.086 mg/mL, which is close to the IC_50_ of vitamin C, measured at 0.032 mg/mL. This comparison highlights the substantial ABTS radical scavenging activity of ABPs, positioning them as potent ABTS radical scavengers suitable for applications requiring strong antioxidant capabilities.

Figure 6C illustrates that the chelation of Fe^2+^ by ABPs exhibits a quadratic correlation between their concentration (*x*) and scavenging capacity (*y*), with statistical significance (*p* < 0.05). The connection is represented by the quadratic formula y = −8.57*x*^2^ + 51.76*x* + 9.76, and it has an *R*^2^ value of 0.967. While ABPs exhibited a higher IC_50_ value of 0.707 mg/mL relative to the positive control, EDTA (IC_50_ = 0.102 mg/mL), their peak scavenging capacity achieved 86.12% of EDTA’s performance. These results underscore the robust chelating activity of ABPs and suggest their significant potential as effective chelators in applications requiring strong metal ion binding.

Figure 6D illustrates that the reducing power (*y*) of ABPs shows a linear correlation with concentration (x), which is statistically significant (*p* < 0.05). This relationship is quantified by the linear equation *y* = 0.232*x* + 0.0848, with an *R*^2^ value of 0.986. When ABPs are at a concentration of 4.0 mg/mL, their reducing power hits 0.967, equating to 67.11% of vitamin C’s effectiveness. This highlights ABPs’ strong electron and hydrogen donation abilities, indicating their potential as catalysts in ending radical chain reactions, a vital part of antioxidant processes.

### 2.6. α-Glucosidase and α-Amylase Inhibitory Capacities of ABPs

**Figure 7:**
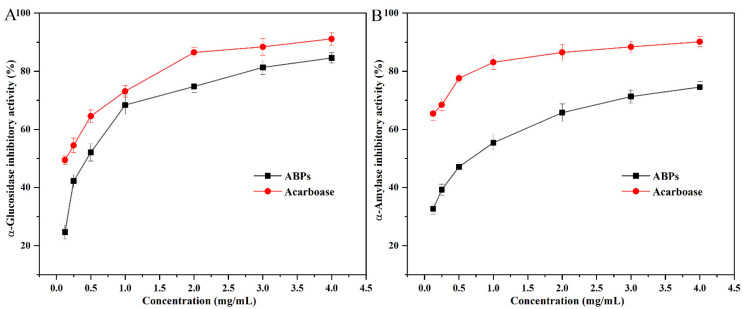
Inhibition rates of ABPs at different concentrations on α-glucosidase (**A**) and α-amylase (**B**) activity.

Digestive enzymes such as α-glycosidase and α-amylase are vital for converting carbohydrates into monosaccharides within the small intestine. Inhibiting these enzymes represents a promising approach to preventing type 2 diabetes [33]. Figure 7A illustrates that ABPs and acarbose suppress α-glucosidase activity in a dose-dependent fashion, with concentrations spanning from 0.125 to 4.0 mg/mL. The suppression effect of ABPs exhibits a concentration-dependent quadratic trend, which is statistically significant (*p* < 0.05) and can be represented by the equation *y* = −4.98*x*^2^ + 33.08*x* + 30.71, with an *R*^2^ of 0.938. The IC_50_ value for ABPs against α-glucosidase is 0.437 mg/mL, which, although higher than acarbose’s 0.170 mg/mL, still demonstrates potential as an antidiabetic agent with minimal side effects and multiple benefits. Figure 7B shows that the inhibition of α-amylase by ABPs increases with concentration. This relationship is also modeled by a quadratic equation, y = −3.53*x*^2^ + 24.22*x* + 33.02, with an *R*^2^ of 0.977. The IC_50_ value for α-amylase inhibition by ABPs is 0.570 mg/mL. Although this is less effective than acarbose (IC_50_ = 0.037 mg/mL), it still indicates significant potential. These findings confirm that ABPs are effective inhibitors of both α-amylase and α-glucosidase, highlighting their potential as functional food ingredients for managing diabetes. The likely mechanism of inhibition involves free carboxyl and hydroxyl groups within the polysaccharides, which can bind to amino acid residues in the enzymes, leading to their inactivation [34]. Additionally, polysaccharides may form complexes with enzymes through electrostatic interactions, further enhancing their inhibitory effects [35]. The IR results reveal that ABPs contain carboxylate ions and hydroxyl groups crucial for this inhibitory activity, providing a solid foundation for their use in dietary interventions aimed at managing type 2 diabetes.

## 3. Materials and Methods

### 3.1. Materials and Chemicals

In November 2022, Xingren Jufeng Adlay Seed Co., Ltd. from Guizhou Province, China, supplied air-dried adlay bran, which was kept at 4 °C until needed. TOKYO Chemical Industry Co., Ltd., Tokyo, Japan supplied an extensive array of monosaccharide standards, such as fucose (Fuc), *N*-acetylgalactosamine (GalNAc), rhamnose (Rha), arabinose (Ara), glucosamine (GlcN), galactose (Gal), glucose (Glc), xylose (Xyl), mannose (Man), fructose (Fru), ribose (Rib), galacturonic acid (GalA), glucuronic acid (GlcA), guluronic acid (GulA), and mannuronic acid (ManA), along with ferrozine, 3-phenylphenol, and trifluoroacetic acid. Reagents for antioxidant assays, such as DPPH (1,1-diphenyl-2-picrylhydrazyl), ABTS (2,2′-azinobis(3-ethylbenzothiazoline-6-sulphonic acid)), and 2,4,6-tris(2-pyridyl)-s-triazine, were obtained from Sigma Chemical Co. in Saint Louis, MO, USA. The pullulan polysaccharide calibration kit was provided by Agilent Co. (Santa Clara, CA, USA). Enzymes α-glucosidase and α-amylase, as well as acarbose and α-d-pyran glucoside (PNPG), were purchased from Yeyuan Biotech Co., Ltd. (Shanghai, China). Aladdin Industrial Inc. (Shanghai, China) supplied the bovine serum albumin. Every other chemical and reagent employed was of analytical quality.

### 3.2. Hot Water Extraction Process of ABPs

Prior to extraction, adlay bran was finely ground and passed through a 40-mesh sieve. The powders underwent defatting with petroleum ether and 95% ethanol at 65 °C for 24 h in a Soxhlet extractor, eliminating small impurities like oligosaccharides, monosaccharides, polyphenols, and pigments. After treatment, the residues were filtered and dried in the air at 50 °C prior to extracting polysaccharides. The extraction process involved a water-to-material ratio ranging from 15 to 35 mL/g, temperatures between 50 and 90 °C, and a duration of 1 to 3 h in a water bath. After extraction, the mixture was centrifuged to separate the supernatant from the residual solids, which were then re-extracted. The solutions were merged and reduced to a third of their initial volume with a rotary evaporator at 54 °C under vacuuming. To precipitate polysaccharides, ethanol was added in a ratio of four parts to one, and the solution was left at 4 °C for 12 h. The resulting precipitates were gathered by centrifuging at 4000 rpm for 10 min, then dissolved again in distilled water and deproteinized with Sevag reagent (chloroform-to-*n*-butanol ratio of 4:1, *v*/*v*) [36]. The mixtures were subsequently dialyzed in distilled water for 72 h using dialysis membranes with a molecular weight cutoff range of 8000 to 14,000 Daltons. The dialyzed solutions were finally freeze-dried to obtain partially purified ABPs. The extraction efficiency was calculated with this equation:

(2) $$\mathrm{Yield}\;(\%,\;w/w)=\frac{\mathrm{weight}\;\mathrm{of}\;\mathrm{dried}\;\mathrm{ABPs}\;(g)}{\mathrm{weight}\;\mathrm{of}\;\mathrm{pretreated}\;\;\mathrm{adlay}\;\mathrm{bran}\;\mathrm{powder}\;(g)}\times 100$$

### 3.3. Optimization via the Response Surface Methodology Test

In this study, the optimization of ABP extraction was conducted using the response surface methodology (RSM) with a Box–Behnken design (BBD) involving three variables at three levels. The extraction parameters when adjusted included extraction temperature (*X*_1_, 70–90 °C), extraction time (*X*_2_, 2–3 h), and the water-to-raw-material ratio (*X*_3_, 25–35 mL/g). This experimental design included 17 randomly executed runs. The analysis involved fitting the data to a second-order polynomial model via a comprehensive quadratic equation, aiming to elucidate the relationships between the variables and the yield of ABPs. The mathematical representation of the model is as follows:

(3) $$Y=\beta 0+\sum_{i=1}^{3}\beta_{i}X_{i}+\sum_{i=1}^{3}\beta_{i}X_{i}^{2}\sum_{i=1}^{2}\sum_{j=i+1}^{3}\beta ijXiXj$$

In this equation, *Y* represents the predicted yield, *β*_0_ is the intercept, and *β_i_*, *β_ii_*, and *β_ij_* are the coefficients for the linear, quadratic, and interaction terms, respectively, with *X_i_* and *X_j_* as the independent variables. The model’s effectiveness was evaluated by assessing the coefficient of correlation (*R*^2^) and performing an analysis of variance (ANOVA). Further analysis involved comparing the coefficient of determination (*R*^2^), adjusted coefficient of determination (*R*^2^_adj_), and predicted coefficient of determination (*R*^2^*_pred_*). The *F*-test verified the significance of the regression coefficients, which is essential for confirming the model’s predictive accuracy.

### 3.4. Structural Characterization of ABPs

#### 3.4.1. Chemical Composition and Molecular Weight Analysis

A neutral sugar content was quantified using the phenol-sulfuric acid method [37], with glucose as the calibration standard. Protein concentrations were determined using the Bradford method [38], with bovine serum albumin (BSA) as the reference standard. Uronic acid levels were measured using the *m*-hydroxydiphenyl colorimetric method [39], with galacturonic acid as the standard.

Molecular weight (Mw) analysis for ABPs was performed using HPGPC on a Shimadzu LC-2010A HPLC system. This process employed a TSK-Gel GMPWXL column (7.8 × 300 mm) and a refractive index (RI) detector. The mobile phase was 0.1 M sodium nitrate solution. Sample was prepared by dissolving it to a concentration of 4 mg/mL and filtering through a 0.22 μm filter. A 20 μL sample was then injected into the HPLC system. Mw was determined from a standard curve generated using pullulan polysaccharide standards, enabling precise molecular sizing.

#### 3.4.2. Monosaccharide Composition Analysis

The monosaccharide composition of ABPs was analyzed using HPAEC following our previous methods [1]. Initially, 5 mg of ABPs was dissolved in 4 mL of 3 mol/L trifluoroacetic acid (TFA) in a sealed tube and hydrolyzed at 120 °C for 3 h. After hydrolysis, the TFA was removed by steam distillation under reduced pressure. The hydrolyzed sample was filtered through a 0.22 μm nylon membrane into a 50 mL vial for chromatographic analysis. The separation process was conducted using a Dionex ICS 6000 ion chromatography system from Thermo Fisher (Waltham, MA, USA), equipped with an electrochemical detector and a Dionex CarboPac PA20 column (3 × 150 mm) stabilized at 30 °C. The mobile phase consisted of three components: A (water), B (15 mmol/L sodium hydroxide), and C (15 mmol/L sodium hydroxide with 100 mmol/L sodium acetate), delivered at a flow rate of 0.3 mL/min. Monosaccharide standards were prepared at concentrations ranging from 1 to 10 ppm and analyzed under the same chromatographic conditions to establish a reference for comparison. The composition of monosaccharides in ABPs was meticulously analyzed by comparing the retention times and peak areas with known standards during chromatographic analysis.

#### 3.4.3. Methylation Analysis

Methylation analysis of ABPs was performed using the Hakomori method [40], which is essential for identifying the positions of glycosidic linkages within the polysaccharides. Initially, ABPs were treated with sodium hydroxide and iodomethane in anhydrous dimethyl sulfoxide (DMSO) to achieve methylation. The completeness of methylation was confirmed by FT-IR analysis, indicated by the disappearance of the hydroxyl (-OH) absorption peak. After methylation, the polysaccharides were hydrolyzed with TFA, reduced with sodium borohydride (NaBH_4_), and acetylated with acetic anhydride, forming partially methylated alditol acetates (PMAAs). These PMAAs were meticulously analyzed using GC-MS. This analysis was conducted on a Shimadzu GCMS-QP2010 system, which was equipped with an Agilent DB-5MS capillary column (30 m × 0.25 mm × 0.25 μm). High-purity helium was used as the carrier gas, with settings adjusted to a split ratio of 5:1 and an injection volume of 1.0 μL. The thermal profile started at 120 °C, rising incrementally to 250 °C at a rate of 3 °C/min, where it was held for 5 min. The analysis was performed in SCAN mode, covering a mass-to-charge (*m*/*z*) range of 30 to 550, to thoroughly evaluate the methylation characteristics of the polysaccharides.

#### 3.4.4. FT-IR Analysis

In the FT-IR analysis, 2 mg of ABPs was mixed with 300 mg of potassium bromide (KBr) and compressed into pellets. The pellets were then analyzed using a Spectrum Two spectrometer (PerkinElmer, Galveston, TX, USA). The spectral analysis covered a wavelength range from 400 to 4000 cm^−1^, with air as the reference medium.

#### 3.4.5. Thermal Analysis

The thermal stability of ABPs was evaluated using thermogravimetric analysis (TGA) on a DTG-60A instrument from Shimadzu Co., Kyoto, Japan. The ABPs were placed in a platinum crucible and analyzed under a nitrogen atmosphere at a constant flow rate of 50 mL/min. The temperature was increased at a controlled rate of 10 °C/min, from 25 °C to 800 °C. This method provided a detailed assessment of the thermal degradation properties of ABPs, revealing their stability and decomposition patterns under specified conditions.

#### 3.4.6. XRD Analysis

XRD analyses of ABPs were performed using a D8 Advance diffractometer (Bruker, Leipzig, Germany). Scans were conducted at a rate of 10 °/min with an incident current of 40 mA, covering a 2θ range of 5 to 80°.

#### 3.4.7. SEM Analysis

Surface morphologies of ABPs were analyzed using a SIGMA 300 SEM from ZEISS, Oberkochen, Germany. Samples were affixed to a stub using double-sided conductive carbon tape for examination. Images were captured at an accelerating voltage of 5.0 kV.

#### 3.4.8. AFM Analysis

Sample preparation for AFM followed a modified method from Yao et al. [41]. ABPs were diluted to 1 μg/mL in Milli-Q water and heated at 50 °C for 2 h to obtain a homogeneous dispersion. A 10 μL aliquot of the dispersion was placed onto a freshly cleaved mica sheet and dried overnight at 25 °C. The nanostructure was imaged using a Dimension ICON AFM (Bruker) equipped with a Si3N4 probe. Measurements were taken at room temperature under ambient conditions using the Tapping mode. NanoScope Analysis software (version 1.8) was used for the analysis, offering detailed insights into the nano-scale structure of the samples, crucial for applications where surface interactions are key.

### 3.5. Rheological Measurement

Steady-shear flow measurements of ABPs at various concentrations (1.0, 2.0, 3.0, 4.0, and 5.0 wt%) were performed using an MCR 302e hybrid rheometer (Anton Paar, Graz, Austria) with a parallel plate geometry (50 mm diameter, 1.0 mm gap). The measurements covered a shear rate range from 0.1 to 1000 s^−1^, maintained at a constant temperature of 25 °C. Each sample was equilibrated at 25 °C for 2 min before testing to ensure uniformity, and all tests were repeated three times to verify accuracy. The resulting data on apparent viscosity (*η*) and shear rate ($\dot{\gamma }$) were analyzed using two distinct rheological models: Cross (Equation (4)) and Carreau (Equation (5)) [42]:

(4) $$\eta =\eta_{\infty }+\frac{\left(\eta_{0}-\eta_{\infty }\right)}{\left[1+{\left(a\dot{\gamma }\right)}^{d}\right]}$$

(5) $$\eta =\eta_{\infty }+\frac{\left(\eta_{0}-\eta_{\infty }\right)}{{\left[1+{\left(c\dot{\gamma }\right)}^{2}\right]}^{p}}$$

In these equations, *η* represents the apparent viscosity (Pa·s), $\eta_{0}$ indicates the zero shear viscosity (Pa·s), and $\eta_{\infty }$ represents the viscosity at an infinite shear rate (Pa·s). The shear rate ($\dot{\gamma }$) is measured in s^−1^. Parameters *a* and *c* are time constants (s), and *d*, *p*, and *n* represent the rate indices and the flow behavior index, respectively. These models are crucial for elucidating the flow properties of ABPs under various conditions, essential for optimizing their application in food products.

### 3.6. Assay of Antioxidant Activity In Vitro of ABPs

ABPs were assessed for antioxidant activity using a range of assays, including DPPH and ABTS radical scavenging, Fe^2+^ chelation, and reducing power. These tests were conducted based on methodologies from our previous studies [21,43], albeit with some modifications to tailor the experiments to the specific characteristics of ABPs.

#### 3.6.1. DPPH Radical Scavenging Activity

In this assay, 100 μL of ABPs at concentrations ranging from 0.125 to 4 mg/mL was mixed with 50 μL of a 0.4 mmol/L ethanolic DPPH solution. The mixture was thoroughly agitated and left to stand for 30 min in the dark at room temperature. Absorbance was measured at 517 nm using a microplate reader, with vitamin C as a positive control. The DPPH radical scavenging activity was calculated using the following equation:

(6) $$\mathrm{Scavenging}\;\mathrm{rate}\;(\%)=(1-\frac{A_{1}-A_{2}}{A_{0}})\;\times \;100$$

where A_0_ is the absorbance of the control (DPPH solution without ABPs), A_1_ is the absorbance after reaction with the ABPs, and A_2_ is the absorbance of the solution containing the ABPs and ethanol.

#### 3.6.2. ABTS Radical Scavenging Activity

The ABTS solution was prepared by mixing 5 mL of 7 mmol/L ABTS salt with 1 mL of 15 mmol/L potassium persulfate and allowing it to react in the dark at room temperature for 24 h. The solution was then diluted to an absorbance of 0.700 ± 0.002 at 734 nm. For testing, 0.2 mL of the solution was mixed with 0.05 mL of ABPs at various concentrations (0.025–0.4 mg/mL) and incubated for 15 min at 20 °C. Vitamin C was used as a reference, and absorbance was measured at 734 nm. The calculation of ABTS scavenging activity follows the same formula as DPPH.

#### 3.6.3. Fe^2+^ Chelating Ability

For the Fe^2+^ chelation assay, 100 μL of ABPs at concentrations from 0.125 to 4 mg/mL was mixed with 20 μL of 2 mmol/L FeCl_2_ and 135 μL of distilled water, followed by 50 μL of 5 mmol/L ferrozine. The mixture was incubated for 10 min at 25 °C, and absorbance was measured at 562 nm, with EDTA as the standard. The Fe^2+^ chelation efficiency was calculated using the same formula as the DPPH radical scavenging assays.

#### 3.6.4. Reducing Power

A solution of 2 mL ABPs at concentrations between 0.125 and 0.4 mg/mL was mixed with 2 mL of 1% potassium ferricyanide and heated for 20 min at 50 °C. Then, 1 mL of 10% trichloroacetic acid was added, and the mixture was centrifuged at 4000 rpm for 10 min. We combined 2 mL of the clear supernatant with 2 mL of distilled water and 0.4 mL of 0.1% FeCl_3_. Absorbance was measured at 700 nm, with vitamin C as the positive control. Reducing power was calculated by measuring the reduction of Fe^3+^ to Fe^2+^. The reducing power is expressed in Equation (7):

(7) $$\mathrm{Reducing}\;\mathrm{power}\;=\;A_{s}\;-\;A_{0}$$

where A_s_ is the measured absorbance of the ABP solution and A_0_ is the baseline absorbance using distilled water in place of the FeCl_3_ solution.

### 3.7. Assay of Hypoglycemic Activity In Vitro of ABPs

#### 3.7.1. α-Glucosidase Inhibitory Activity

The α-glucosidase inhibitory activity of ABPs was evaluated using a modified protocol based on previous research [20]. Initially, 150 μL of α-glucosidase solution (0.4 U/mL in 0.1 M phosphate buffer, pH 6.9) was combined with 100 μL of ABPs at different concentrations (ranging from 0.125 to 4.0 mg/mL). The solution was subsequently kept at 37 °C for a duration of 10 min. Subsequently, 100 μL of a 7.5 mM pNPG solution was added as the substrate. Following another 20 min of incubation at 37 °C, the reaction was halted by introducing 1 mL of 1 mol/L sodium carbonate. The absorbance at 405 nm was recorded with a microplate reader. Acarbose served as the reference standard. The inhibitory effect was determined by applying this equation:

(8) $$\alpha -\mathrm{Glucosidase}\;\mathrm{inhibitory}\;\mathrm{rate}\;(\%)=(1-\frac{A_{1}-A_{2}}{A_{0}})\;\times \;100$$

where A_0_ represents the absorbance in the absence of enzyme activity (control with just the solvent), A_1_ indicates the absorbance with both the test sample and enzyme, and A_2_ signifies the absorbance corresponding to full enzyme activity (control with both solvent and enzyme).

#### 3.7.2. α-Amylase Inhibitory Activity

The α-amylase inhibitory activity of ABPs was evaluated using a modified version of a previously established method [44]. ABP solutions with concentrations ranging from 0.125 to 4.0 mg/mL were prepared in 0.1 mol/L phosphate buffer (PBS, pH 6.8). Simultaneously, solutions of 1% (*w*/*v*) soluble starch and 6 mmol/L sodium chloride were prepared using the same buffer. During the assay, 500 µL of the starch solution (2 mg/mL) was mixed with 20 µL of α-amylase solution (8 U/mL) and 20 µL of ABP solution. The mixture was incubated at 25 °C for 10 min to allow the enzymatic reaction to proceed. Next, 20 µL of the mixture was mixed with 80 µL of dinitrosalicylic acid (DNS) and then heated at 100 °C for 5 min. The absorbance of the final mixture was measured at 540 nm, with acarbose used as the positive control. The inhibition efficacy of ABPs on α-amylase was calculated using the same formula as that used for the α-glucosidase inhibitory activity. A_0_ is the absorbance of the control mixture containing α-amylase and starch. A_1_ is the absorbance of the test mixture, which includes α-amylase, starch, and ABPs. A_2_ is the absorbance of the mixture containing only ABPs and starch.

### 3.8. Statistical Analysis

To ensure data consistency and reliability across the study, each experiment was conducted in triplicate, with the results reported as the mean ± standard deviation (SD). For regression analysis and graphical optimization, Design Expert software, version 12 (Stat-Ease, Minneapolis, MN, USA), was employed. Statistical analyses, including ANOVA and a subsequent Duncan’s post hoc test, were performed using SPSS version 22 (SPSS Inc., Chicago, IL, USA). For the visual representation of the data, which aids in the effective illustration and interpretation of findings, Origin 2021 software (Origin Lab Corp., Northampton, MA, USA) was utilized.

## 4. Conclusions

This study provides the first comprehensive investigation into the extraction, characterization, antioxidant, and hypoglycemic activities of polysaccharides derived from adlay bran, a by-product of adlay processing. By employing a hot water extraction technique refined using the response surface methodology (RSM), we determined the best extraction parameters to be a temperature of 80.0 °C, a duration of 2.5 h, and a water-to-material ratio of 31.6 mL/g. This approach yielded 2.61 ± 0.07%, closely aligning with the predicted outcomes. The polysaccharides, termed ABPs, are acidic, polydisperse heteropolysaccharides predominantly composed of glucose, with molecular weights of 12.88 and 2.31 kDa. ABPs mainly contain eight kinds of linkage-type units: →3,4)-Rha*p*-(1→ at 5.52%, →4)-Glc*p*-(1→ at 25.64%, Glc*p*-(1→ at 9.70%, →3,4)- Glc*p*-(1→ at 19.11%, →4)-Xyl*p*-(1→ at 7.05%, →3)-Glc*p*-(1→ at 13.23%, →3,4)-Gal*p*-(1→ at 9.26%), and →4,6)-Gcl*p*-(1→ at 12.49%. Thermal analysis confirmed the robust stability of ABPs, while rheological evaluations revealed their characteristic shear-thinning behavior and viscoelastic properties, accurately modeled by the Carreau equation. In biochemical assays, ABPs demonstrated potent antioxidant activities—evidenced by DPPH and ABTS radical scavenging—and significant metal chelating capacity and reducing power. Additionally, ABPs effectively inhibited α-amylase and α-glucosidase, underscoring their potential as functional food additives and pharmaceutical agents. Despite these promising results, the specific mechanisms underlying ABPs’ antioxidant and hypoglycemic effects, and their relationship with the polysaccharides’ physicochemical properties and structural features, remain to be elucidated. Ongoing studies aim to clarify these molecular mechanisms, enhancing our understanding of ABPs’ potential to valorize adlay bran. This research serves as a vital reference for leveraging the value-added properties of this agricultural by-product.

## Figures and Tables

**Table 1:** The Box–Behnken design (BBD) and the results for the yield of ABPs.

Std. Order	*X*_1_ (Extraction Temperature, °C)	*X*_2_ (Extraction Time, h)	*X*_3_ (Ratio of Water to Raw Material, mL/g)	Extraction Yield (%)
1	−1 (70)	−1 (2)	0 (30)	1.61
2	1 (90)	−1 (2)	0 (30)	1.82
3	−1 (70)	1 (3)	0 (30)	1.75
4	1 (90)	1 (3)	0 (30)	1.72
5	−1 (70)	0 (2.5)	−1 (25)	1.69
6	1 (90)	0 (2.5)	−1 (25)	1.91
7	−1 (70)	0 (2.5)	1 (35)	2.05
8	1 (90)	0 (2.5)	1 (35)	1.93
9	0 (80)	−1 (2)	−1 (25)	2.23
10	0 (80)	1 (3)	−1 (25)	1.80
11	0 (80)	−1 (2)	1 (35)	1.98
12	0 (80)	1 (3)	1 (35)	2.30
13	0 (80)	0 (2.5)	0 (30)	2.55
14	0 (80)	0 (2.5)	0 (30)	2.56
15	0 (80)	0 (2.5)	0 (30)	2.54
16	0 (80)	0 (2.5)	0 (30)	2.51
17	0 (80)	0 (2.5)	0 (30)	2.55

**Table 2:** Analysis of variance (ANOVA) for response surface quadratic model.

Source	Sum of Squares	df	Mean Square	*F*-Value	*p*-Value	
Model	1.95	9	0.2168	210.61	<0.0001	Significant
*X*_1_	0.0098	1	0.0098	9.52	0.0177	Significant
*X*_2_	0.0006	1	0.0006	0.5951	0.4657	
*X*_3_	0.0496	1	0.0496	48.20	0.0002	Significant
*X*_1_ *X*_2_	0.0144	1	0.0144	13.99	0.0073	Significant
*X*_1_ *X*_3_	0.0289	1	0.0289	28.08	0.0011	Significant
*X*_2_ *X*_3_	0.1406	1	0.1406	136.62	<0.0001	Significant
*X*_1_^2^	1.05	1	1.05	1021.66	<0.0001	Significant
*X*_2_^2^	0.4238	1	0.4238	411.72	<0.0001	Significant
*X*_3_^2^	0.0913	1	0.0913	88.70	<0.0001	Significant
Residual	0.0072	7	0.0010			
Lack of Fit	0.0057	3	0.0019	5.16	0.0735	Not significant
Pure Error	0.0015	4	0.0004			
Std. Dev.	0.0321					
Mean	2.09					
*R* ^2^	0.9963					
Adjusted *R*^2^	0.9916					
Predicted *R*^2^	0.9520					
C.V. %	1.54					

**Table 3:** Chemical composition and monosaccharide composition, and molecular weight of ABPs.

Chemical Composition (%, g/g)	ABPs
Neutral sugar	84.12 ± 1.55
Protein	2.32 ± 0.43
Uronic acid	3.03 ± 0.37
Monosaccharide composition (molar%)	
Rha	2.12 ± 0.04
Ara	2.40 ± 0.08
GlcN	0.52 ± 0.01
Gal	7.94 ± 0.23
Glc	77.12 ± 1.47
Xyl	3.51 ± 0.67
Man	2.55 ± 0.59
GlcA	3.82 ± 0.44
Molecular weight	
Peak 1 (kDa)	12.88 ± 1.55
Peak 2 (kDa)	2.31 ± 0.17

**Table 4:** Results of methylation analysis for ABPs.

RT (min)	Methylated Alditol Acetate	Major Mass Fragments (*m*/*z*)	Molar Ratio (%)	Type of Linkage
14.506	2-Me_1_-Rha	43, 87, 99, 113, 117, 129, 141, 159, 173	3.52	→3,4)-Rha*p*-(1→
15.761	2,3,6-Me_3_-Glc	43, 87, 99, 101, 113, 117, 129, 131, 161, 173, 233	25.64	→4)-Glc*p*-(1→
15.99	2,3,4,6-Me_4_-Glc	43, 71, 87, 101, 117, 129, 145, 161, 205	9.70	Glc*p*-(1→
19.163	2,6-Me_2_-Glc	43, 87, 97, 117, 159, 185	19.11	→3,4)-Glc*p*-(1→
19.755	2,3-Me_2_-Xyl	43, 71, 87, 99, 101, 117, 129, 161, 189	7.05	→4)-Xyl*p*-(1→
22.114	2,4,6-Me_3_-Glc	43, 87, 99, 101, 117, 129, 161, 173, 233	13.23	→3)-Glc*p*-(1→
23.911	2,6-Me_2_-Gal	43, 87, 99, 117, 129, 143, 159	9.26	→3,4)-Gal*p*-(1→
25.341	2,3-Me_2_-Glc	59, 102, 118, 142, 201, 231, 261	12.49	→4,6)-Gcl*p*-(1→

**Table 5:** Cross and Carreau–Yasuda model parameters at different ABP concentrations.

ABPs (mg/mL)	Cross Model Fitting Parameters					Carreau Model Fitting Parameters				
	*a* (s)	*d*	*η_0_* (Pa·s)	*η_∞_* (Pa·s)	*R* ^2^	*c* (s)	*p*	*η_0_* (Pa·s)	*η_∞_* (Pa·s)	*R* ^2^
10	4.1190	0.7669	0.0074	0.0026	0.9875	34.1964	0.0469	0.0127	0.0025	0.9811
20	4.7690	0.5646	3.8333	0.0047	0.9833	31.6451	0.1338	0.0143	0.0046	0.9804
30	5.0450	0.5276	9.2999	0.0069	0.9885	18.3896	0.4247	0.0273	0.0068	0.9821
40	6.0632	0.4810	9.6951	0.0138	0.9987	1.2686	0.7399	0.0156	0.0122	0.9931
50	16.6411	0.2868	21.2648	0.0294	0.9986	0.0001	0.7861	0.1580	0.0216	0.9950

## Data Availability

Data are contained within this article.

## Supplementary Materials

The following supporting information can be downloaded at https://www.mdpi.com/article/10.3390/molecules29194707/s1, Figure S1: SEM images of ABPs ((A): magnification 50×, (B): magnification 200×); Figure S2: AFM images of ABPs.

## References

[1] Chen K., Zhang Q., Yang S., Zhang S., Chen G. (2024). Comparative study on the impact of different extraction technologies on structural characteristics, physicochemical properties, and biological activities of polysaccharides from seedless chestnut rose (*Rosa sterilis*) fruit. Foods.

[2] He X., Fan H., Sun M., Li J., Xia Q., Jiang Y., Liu B. (2024). Chemical structure and immunomodulatory activity of a polysaccharide from Saposhnikoviae Radix. Int. J. Biol. Macromol..

[3] Li S., Zhao Z., He Z., Yang J., Feng Y., Xu Y., Wang Y., He B., Ma K., Zheng Y. (2024). Effect of structural features on the antitumor activity of plant and microbial polysaccharides: A review. Food Biosci..

[4] Li Q.Y., Dou Z.M., Duan Q.F., Chen C., Liu R.H., Jiang Y.M., Yang B., Fu X. (2024). A comparison study on structure-function relationship of polysaccharides obtained from sea buckthorn berries using different methods: Antioxidant and bile acid-binding capacity. Food Sci. Hum. Wellness.

[5] Zhao L.C., Wu L.B., Li L.Q., Zhu J., Chen X., Zhang S.Y., Li L., Yan J.K. (2023). Physicochemical, structural, and rheological characteristics of pectic polysaccharides from fresh passion fruit (*Passiflora edulis f. flavicarpa* L.) peel. Food Hydrocoll..

[6] Zhu H., Xu L., Wang J., Zhang Z.H., Xu X.H., Yang K., Sun P.L., Liao X.J., Cai M. (2023). Rheological behaviors of ethanol-fractional polysaccharides from *Dendrobium officinale* in aqueous solution: Effects of concentration, temperature, pH, and metal ions. Food Hydrocoll..

[7] Ji X., Guo J., Cao T., Zhang T., Liu Y., Yan Y. (2023). Review on mechanisms and structure-activity relationship of hypoglycemic effects of polysaccharides from natural resources. Food Sci. Hum. Wellness.

[8] Yang M., Ren W., Li G., Yang P., Chen R., He H. (2022). The effect of structure and preparation method on the bioactivity of polysaccharides from plants and fungi. Food Funct..

[9] Ding Y., Zhang G., Ni C., Yu G., Cheng J., Zheng H. (2020). Understanding the mechanism of change in morphological structures, visualization features, and physicochemical characteristics of adlay seeds (*Coix lacryma jobi* L.): The role of heat soaking. J. Cereal Sci..

[10] Devaraj R.D., Jeepipalli S.P.K., Xu B. (2020). Phytochemistry and health promoting effects of Job’s tears (*Coix lacryma-jobi*)—A critical review. Food Biosci..

[11] Wei X., Li Y., Zhou S., Guo C., Dong X., Li Q., Guo J., Wang Y., Huang L. (2023). The Differences of nutrient components in edible and feeding coix seed at different developmental stages based on a combined analysis of metabolomics. Molecules.

[12] Chen H.-J., Shih C.-K., Hsu H.-Y., Chiang W. (2010). Mast cell-dependent allergic responses are inhibited by ethanolic extract of adlay (*Coix lachrymal jobi* L. var*. ma*-*yuen* Stapf) Testa. J. Agric. Food Chem..

[13] Ge Q., Hou C.-l., Rao X.-h., Zhang A.-q., Xiao G.-m., Wang L.-y., Jin K.-n., Sun P.-l., Chen L.-C. (2024). In vitro fermentation characteristics of polysaccharides from coix seed and its effects on the gut microbiota. Int. J. Biol. Macromol..

[14] Chang W.-C., Hu Y.-T., Huang Q., Hsieh S.-C., Ting Y. (2020). Development of a topical applied functional food formulation: Adlay bran oil nanoemulgel. LWT-Food Sci. Technol..

[15] Huang D.-W., Wu C.-H., Shih C.-K., Liu C.-Y., Shih P.-H., Shieh T.-M., Lin C.-I., Chiang W., Hsia S.-M. (2014). Application of the solvent extraction technique to investigation of the anti-inflammatory activity of adlay bran. Food Chem..

[16] Tang X., Wang Z., Zheng J., Kan J., Chen G., Du M. (2023). Physicochemical, structure properties and in vitro hypoglycemic activity of soluble dietary fiber from adlay *(Coix lachryma-jobi* L. var. *ma-yuen* Stapf) bran treated by steam explosion. Front. Nutr..

[17] Zhou S., Tang X., Hegyi F., Nagy A., Takacs K., Zalan Z., Chen G., Du M. (2024). In vitro digestion and fermentation characteristics of soluble dietary fiber from adlay (*Coix lachryma-jobi* L. var. *ma-yuen* Stapf) bran modified by steam explosion. Food Res. Int..

[18] Lin L., Yang Q., Zhao K., Zhao M. (2018). Identification of the free phenolic profile of Adlay bran by UPLC-QTOF-MS/MS and inhibitory mechanisms of phenolic acids against xanthine oxidase. Food Chem..

[19] Li Y., Tian X., Li S., Chang L., Sun P., Lu Y., Yu X., Chen S., Wu Z., Xu Z. (2019). Total polysaccharides of adlay bran (*Coix lachryma-jobi* L.) improve TNF-α induced epithelial barrier dysfunction in Caco-2 cells via inhibition of the inflammatory response. Food Funct..

[20] Zhang Q., Wu S., Dai Q., Hu P., Chen G. (2024). Effects of Different drying methods on the structural characteristics and multiple bioactivities of *Rosa roxburghii* tratt fruit polysaccharides. Foods.

[21] Chen G.J., Chen K.W., Zhang R.F., Chen X.L., Hu P., Kan J.Q. (2018). Polysaccharides from bamboo shoots processing by-products: New insight into extraction and characterization. Food Chem..

[22] Zhao Z.W., Wang L., Ruan Y., Wen C.N., Ge M.H., Qian Y.Y., Ma B.J. (2023). Physicochemical properties and biological activities of polysaccharides from the peel of *Dioscorea opposita* Thunb. extracted by four different methods. Food Sci. Hum. Wellness.

[23] Xiao Z., Yan C., Jia C., Li Y., Li Y., Li J., Yang X., Zhan X., Ma C. (2023). Structural characterization of chia seed polysaccharides and evaluation of its immunomodulatory and antioxidant activities. Food Chem.-X.

[24] Jiao X., Li F., Zhao J., Wei Y., Zhang L., Wang H., Yu W., Li Q. (2023). Structural diversity and physicochemical properties of polysaccharides isolated from pumpkin (*Cucurbita moschata*) by different methods. Food Res. Int..

[25] Bai C., Chen R., Zhang Y., Bai H., Tian L., Sun H., Li D., Wu W. (2023). Comparison in structural, physicochemical and functional properties of sweet potato stems and leaves polysaccharide conjugates from different technologies. Int. J. Biol. Macromol..

[26] Reis S.E., Andrade R.G.C., Accardo C.M., Maia L.F., Oliveira L.F.C., Nader H.B., Aguiar J.A.K., Medeiros V.P. (2020). Influence of sulfated polysaccharides from *Ulva lactuca* L. upon Xa and IIa coagulation factors and on venous blood clot formation. Algal Res. -Biomass Biofuels Bioprod..

[27] Liu J., Pu Q., Qiu H., Di D. (2021). Polysaccharides isolated from *Lycium barbarum* L. by integrated tandem hybrid membrane technology exert antioxidant activities in mitochondria. Ind. Crops Prod..

[28] Qi X., Liu R., Chen M., Li Z., Qin T., Qian Y., Zhao S., Liu M., Zeng Q., Shen J. (2019). Removal of copper ions from water using polysaccharide-constructed hydrogels. Carbohydr. Polym..

[29] Gong Y., Ma Y., Cheung P.C.-K., You L., Liao L., Pedisic S., Kulikouskaya V. (2021). Structural characteristics and anti-inflammatory activity of UV/H_2_O_2_-treated algal sulfated polysaccharide from *Gracilaria lemaneiformis*. Food Chem. Toxicol..

[30] Liang Z., Yin Z., Liu X., Ma C., Wang J., Zhang Y., Kang W. (2022). A glucomannogalactan from *Pleurotus geesteranus*: Structural characterization, chain conformation and immunological effect. Carbohydr. Polym..

[31] Xu G.-Y., Liao A.-M., Huang J.-H., Zhang J.-G., Thakur K., Wei Z.-J. (2019). The rheological properties of differentially extracted polysaccharides from potatoes peels. Int. J. Biol. Macromol..

[32] Kumar Y., Roy S., Devra A., Dhiman A., Prabhakar P.K. (2021). Ultrasonication of mayonnaise formulated with xanthan and guar gums: Rheological modeling, effects on optical properties and emulsion stability. LWT-Food Sci. Technol..

[33] Wang L., Zhang B., Xiao J., Huang Q., Li C., Fu X. (2018). Physicochemical, functional, and biological properties of water-soluble polysaccharides from *Rosa roxburghii* Tratt fruit. Food Chem..

[34] Xue H., Hao Z., Gao Y., Cai X., Tang J., Liao X., Tan J. (2023). Research progress on the hypoglycemic activity and mechanisms of natural polysaccharides. Int. J. Biol. Macromol..

[35] Xu R., Zheng L., Su G., Zhao M., Yang Q., Wang J. (2022). Electrostatic interactions with anionic polysaccharides reduced the degradation of pepsin soluble undenatured type II collagen during gastric digestion under pH 2.0. Food Hydrocoll..

[36] Sevag M.G., Lackman D.B., Smolens J. (1938). The isolation of the components of streptococcal nucleoproteins in serologically active form. J. Biol. Chem..

[37] Dubois M., Gilles K.A., Hamilton J.K., Rebers P.A., Smith F. (1956). Colorimetric method for determination of sugars and related substances. Anal. Chem..

[38] Hildebrandt S., Steinhart H., Paschke A. (2008). Comparison of different extraction solutions for the analysis of allergens in hen’s egg. Food Chem..

[39] Blumenkr N., Asboehan G. (1973). New method for quantitative-determination of uronic acids. Anal. Biochem..

[40] Hakomori S.I. (1964). Rapid permethylation of glycolipid polysaccharide catalyzed by methylsulfinyl carbanion in dimethyl sulfoxide. J. Biochem..

[41] Yao J., Yang C., Shi K., Liu Y., Xu G., Pan S. (2023). Effect of pulp cell wall polysaccharides on citrus fruit with different mastication traits. Food Chem..

[42] Meng Q., Wang Q., Chen L., Li J.W., Fan L.P., Gu Z.H., Shi G.Y., Ding Z.Y. (2023). Rheological properties and thickening effect of high molecular weight exopolysaccharide and intracellular polysaccharide from *Schizophyllum commune*. Food Hydrocoll..

[43] Chen G.J., Fang C.C., Ran C.X., Tan Y., Yu Q.Q., Kan J.Q. (2019). Comparison of different extraction methods for polysaccharides from bamboo shoots (*Chimonobambusa quadrangularis*) processing by-products. Int. J. Biol. Macromol..

[44] Chen J., Zhou M., Liu M., Bi J. (2022). Physicochemical, rheological properties and in vitro hypoglycemic activities of polysaccharide fractions from peach gum. Carbohydr. Polym..

