# Supplementary material for: Extraction, Structural Characterization, and Physicochemical and Biological Properties of Water-Soluble Polysaccharides from Adlay Bran
Source: Molecules. 2024 Oct 4;29(19):4707. doi: 10.3390/molecules29194707 (PMC11478194; doi:10.3390/molecules29194707)
Supplement: Supplementary file 1 [file molecules-29-04707-s001.zip › molecules-3195439-supplementary.docx]

**Supplementary Materials**


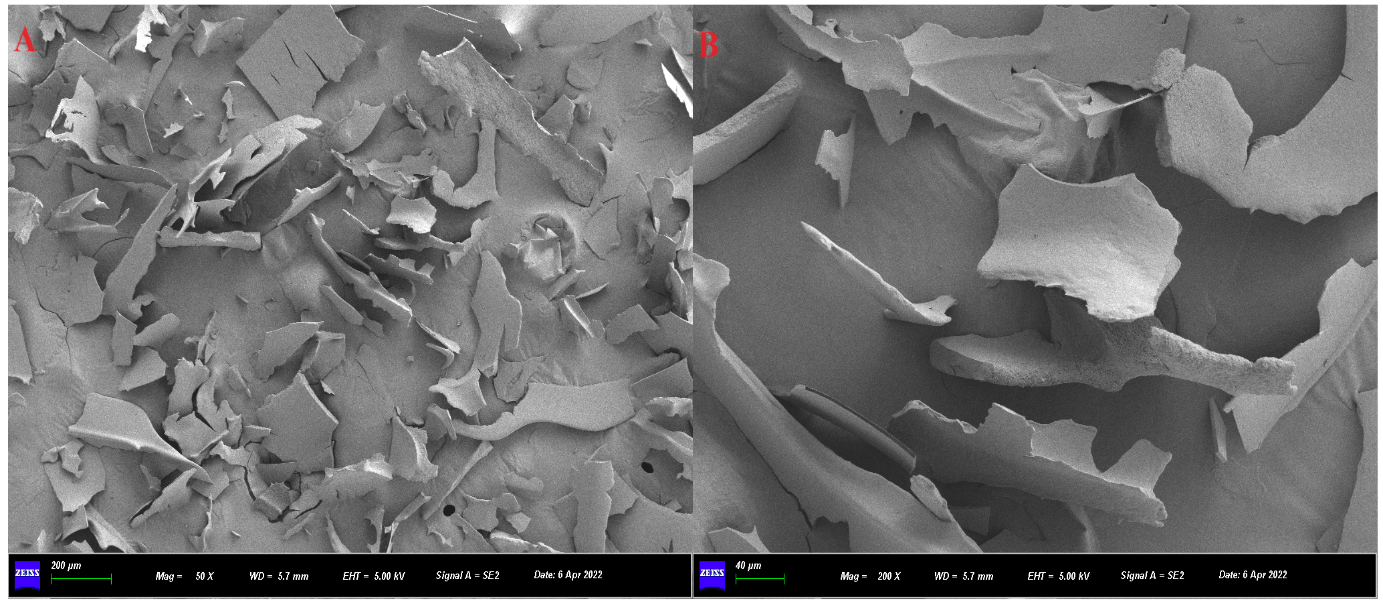


**Figure S1.** SEM images of ABPs. (A: magnification 50×, B: magnification 200×).


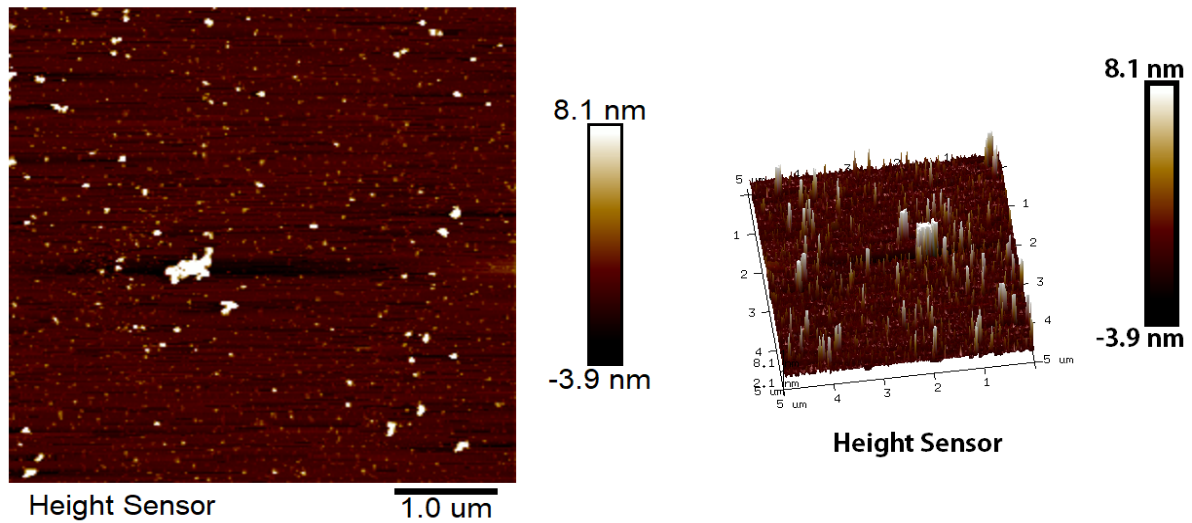


**Figure S2.** AFM images of ABPs.
